# Genome-Wide Association and Two-Sample Mendelian Randomization Analyses of Plasma Ghrelin and Gastrointestinal Cancer Risk

**DOI:** 10.1158/1055-9965.EPI-23-0757

**Published:** 2023-10-04

**Authors:** Susanna C. Larsson, Jonas Höijer, Jing Sun, Xue Li, Stephen Burgess, Karl Michaёlsson

**Affiliations:** 1 Unit of Medical Epidemiology, Department of Surgical Sciences, Uppsala University, Uppsala, Sweden; 2 Unit of Cardiovascular and Nutritional Epidemiology, Institute of Environmental Medicine, Karolinska Institutet, Stockholm, Sweden; 3 Department of Big Data in Health Science, School of Public Health and The Second Affiliated Hospital, Zhejiang University School of Medicine, Hangzhou, China; 4 Department of Public Health and Primary Care, University of Cambridge, Cambridge, United Kingdom; 5 MRC Biostatistics Unit, University of Cambridge, Cambridge, United Kingdom

**Keywords:** Cancer, Genome-wide association study, Ghrelin, Mendelian randomization, Single-nucleotide polymorphisms

## Abstract

**Background:**

Observational studies have suggested that the gut hormone ghrelin is an early marker of future risk of developing gastrointestinal cancer. However, whether ghrelin is a causal risk factor remains unclear. We conducted a genome-wide association study (GWAS) of plasma ghrelin and used Mendelian randomization (MR) to investigate the possible causal association between ghrelin and gastrointestinal cancer risk.

**Methods:**

Genetic variants associated with plasma ghrelin were identified in a GWAS comprising 10 742 Swedish adults in the discovery (N=6259) and replication (N=4483) cohorts. The association between ghrelin and gastrointestinal cancer was examined through a two-sample MR analysis using the identified genetic variants as instruments and GWAS data from the UK Biobank, FinnGen, and a colorectal cancer consortium.

**Results:**

GWAS found associations between multiple genetic variants within ±200 kb of the *GHRL* gene and plasma ghrelin. A two-sample MR analysis revealed that genetically predicted higher plasma ghrelin levels were associated with a lower risk of gastrointestinal cancer in UK Biobank and in a meta-analysis of the two studies. The combined odds ratio per approximate doubling of genetically predicted plasma ghrelin was 0.91 (95% confidence interval, 0.85-0.99; *P*=0.02). Colocalization analysis revealed limited evidence of shared causal variants for plasma ghrelin and gastrointestinal cancer at the *GHRL* locus (posterior probability H_4_=24.5%); however, this analysis was likely underpowered.

**Conclusions:**

Our study provides evidence in support of a possible causal association between higher plasma ghrelin levels and a reduced risk of gastrointestinal cancer.

**Impact:**

Elevated plasma ghrelin levels might reduce the risk of gastrointestinal cancer.

## Introduction

Ghrelin is a 28 amino acid peptide produced by the enteroendocrine cells of the gastrointestinal tract, particularly in the stomach (1–3). In addition to its growth hormone-releasing activity (1), ghrelin regulates appetite, energy homeostasis, gastric acid secretion, and gut motility (4–6). Accumulating evidence further indicates that ghrelin modulates cell proliferation and apoptosis and may affect the development of gastrointestinal cancer (3,5,7,8). Several observational studies have found that low circulating levels of ghrelin are associated with an increased risk of esophageal (9–11), stomach (10,12,13), and colorectal cancers (in the years approaching diagnosis) (14). In contrast, other observational studies reported that low ghrelin levels were associated with a reduced risk of esophageal cancer (13) but were not clearly associated with colorectal cancer (15,16). Given the observational design of the available studies on ghrelin levels and gastrointestinal cancer, it remains unknown whether the reported associations are causal or driven by biases inherited in observational studies, such as confounding and reverse causation.

Mendelian randomization (MR) is an increasingly exploited study design that can improve causal inference in observational studies by leveraging genetic variants that are strongly associated with exposure (e.g., ghrelin) as instruments to decipher the causal effect of exposure on the outcome (e.g., due to the unchangeable nature of genetic variants and the random allocation of alleles at conception). Compared to classical observational studies, MR studies are less susceptible to reverse causation bias and confounding from self-selected behaviors and environmental factors.

First, we conducted a genome-wide association study (GWAS) to identify genetic variants associated with ghrelin. Second, we used genetic variants identified through the two-sample MR framework to investigate the association between lifelong higher ghrelin levels and the risk of gastrointestinal cancer.

## Materials and Methods

### Participants in GWAS analysis

The GWAS was based on data from the Swedish Infrastructure for Medical Population-Based Life-Course and Environmental Research (SIMPLER) (https://www.simpler4health.se/), comprising two large longitudinal cohorts and a biobank. In addition, SIMPLER encompasses two clinical sub-cohorts, each with participants born between 1920-1952 from neighboring Swedish counties. Participants in the clinical subcohorts were randomly chosen from two larger cohorts (i.e., the Swedish Mammography Cohort and Cohort of Swedish Men) and invited to participate in a health examination. Participants from the clinical sub-cohorts with accurate GWAS, of European descent according to GWAS results, and with protein data, were eligible for inclusion in the present GWAS, which encompassed a discovery cohort of 6259 women and men who resided in Västmanland County and provided a blood sample between 2010 and 2019, and a replication cohort of 4483 women who lived in Uppsala County and provided a blood sample between 2003 and 2009.

### Ghrelin measurement

Blood samples were collected in the morning after 12 hours of overnight fasting. After 5-10 min of storage at room temperature, the samples were centrifuged at room temperature at 310 *g* for 11 min, after which the buffy coat was extracted. The samples were immediately centrifuged at 1615 *g* for 11 min at 4 °C. Subsequently, the plasma samples were aliquoted into multiple tubes and stored at −80 °C until analysis. The samples were light-protected from the time of blood collection and sample preparation until freezing.

Total plasma ghrelin concentration (UniProt Q9UBU3) was measured using a high-throughput multiplex immunoassay (Olink Proseek Multiplex CVD II; Olink Bioscience, Uppsala, Sweden), which runs normalized protein expression values on a log_2_ scale standardized for each analysis plate. Olink’s proximity extension assay technology uses pairs of antibodies equipped with DNA reporter molecules to produce DNA amplicons, which are subsequently quantified using the Fluidigm BioMark HD real-time polymerase chain reaction platform (17,18). As only correctly matched antibody pairs produce a signal, the proximity extension assay technique has an accuracy advantage over the conventional multiplex immunoassays. Olink NPX Manager software was used for data analysis. A one-unit increment in NPX corresponds to an approximate doubling of measured plasma ghrelin levels. The within- and between-run precision coefficients of variation were 9% and 16%, respectively. More details on the protein analyses have been reported previously (19).

### Genotyping and GWAS analysis

Details of genotyping and GWAS quality control for the discovery and replication cohorts have been previously described (19). The genetic dataset comprised of ~7.8 million DNA markers. The GWAS analysis was conducted using linear regression in SNPTEST (20), assuming an additive genetic model and with adjustment for age, sex, and five genetic principal components. Genetic variants associated with plasma ghrelin at *P*<5×10^-8^ in the discovery cohort were tested in the replication cohort. Single-nucleotide polymorphisms (SNPs) associated with plasma ghrelin in the same direction (*P*<0.05) in the replication cohort were considered replicated.

### Two-sample MR analysis

The SNPs identified in the GWAS were used as instrumental variables for plasma ghrelin levels. Two sets of genetic instruments were used in this study. We selected SNPs with low LD (*R*^2^<0.1) and within ±200 kb of the *GHRL* gene for the first genetic instrument. SNPs with low LD were identified by clumping (based on Europeans from the 1000 genomes reference panel) implemented using the *TwoSampleMR* package in R (21). We applied the multiplicative random effects inverse-variance weighted method and adjusted for correlations between SNPs (22). The correlation matrix was obtained from 367 643 unrelated adults of European descent in the UK Biobank. As a second genetic instrument, we selected the *cis*-SNP with the strongest association (lowest *P*-value) with plasma ghrelin, and the MR estimate was computed by dividing the beta coefficient for the SNP-outcome association by the beta coefficient for the SNP-ghrelin association.

We used the two-sample MR design to examine the associations of plasma ghrelin proxied by the four and one SNP instruments with any gastrointestinal cancer (primary outcome) and specific cancers in the gastrointestinal tract (ancillary outcomes) using outcome data from the UK Biobank (as described previously (23)) as well as publicly available summary genetic data from the FinnGen study (release R8) (24,25). The association estimates were adjusted for age, sex, and the 10 principal genetic components. The outcome classification is provided in Table S1 and the number of cases for each outcome is shown in Table S2. In the UK Biobank there were 11 952 individuals diagnosed with gastrointestinal cancer, and in FinnGen, there were 9822 such cases. For colorectal cancer, we additionally used summary-level data from a meta-analysis of 16 GWASs, comprising 73 673 cases and 86 854 controls of European ancestries (26). Studies included in the colorectal cancer meta-analysis dataset used in the present study are presented in Table S3.

An online tool was used to calculate the statistical power of the MR analysis (27). MR associations with *P* value < 0.05 were regarded as statistically significant. MR analyses were performed using the *MendelianRandomization* (28) and *TwoSampleMR* (21) packages in R. Meta-analysis of results from the UK Biobank and FinnGen studies was conducted using the *metan* command in Stata (College Station, Texas, USA), and heterogeneity between the two studies was quantified using the *I*^2^ statistic (29).

### Colocalization analysis

Colocalization analysis, using the coloc package in R (30), was conducted as a sensitivity analysis to evaluate whether plasma ghrelin and gastrointestinal cancer share the same causal genetic variant at the *GHRL* locus (±200 kb windows around *GHRL* gene). Such an analysis can indicate whether the phenotypes are influenced by different causal genetic variants that are in LD, indicative of horizontal pleiotropy (i.e., when a genetic variant affects the outcome through a pathway that does not involve the studied exposure) and violation of the exclusion restriction assumption (31). H_4_ >50% was considered supportive of colocalization of the two phenotypes.

### Ethics approval and consent to participate

The studies included in this study were approved by a relevant ethical review authority, and the participants provided written informed consent. The Swedish Ethical Review Authority approved the analyses for this study. All methods were performed in accordance with the relevant guidelines and regulations.

## Results

### GWAS

The mean (± standard deviation) age of participants in the discovery cohort (34.7% women) and replication cohort (100% women) was respectively 73.8 (5.3) and 67.1 (6.8) years and the corresponding body mass index was 26.6 (4.0) kg/m^2^ and 25.9 (4.3) kg/m^2^. In the replication cohort, 28 SNPs around the *GHRL* locus (within ±200 kb of the gene) on chromosome 3 were associated with plasma ghrelin at *P*<5×10^-8^ (Table 1). These SNPs were all associated with plasma ghrelin in the same direction in the replication cohort (*P*<0.001) as well as in both cohorts combined (*P*<9×10^-11^) (Table 1).

### Selection and performance of the two genetic instruments

The first genetic instrument was based on SNPs in low LD and within ±200 kb of the *GHRL* gene. The instrument explained 4.6% of the variance in plasma ghrelin levels in the UK Biobank study when accounting for the correlations among SNPs. The inclusion of more SNPs (in modest to high LD [R2>0.1]) as instrumental variables did not increase the phenotypic variance when accounting for the correlations. Power was high (≥99% to detect a significant OR of 0.8 or 1.2) in MR analysis of any gastrointestinal cancer and colorectal cancer, but low in MR analysis of other specific gastrointestinal cancers (Table S2). The strongest SNP (smallest *P* value), rs34911341 in *GHRL*, was used as a secondary genetic instrument.

### Two-sample MR analysis

**Figure 1:**
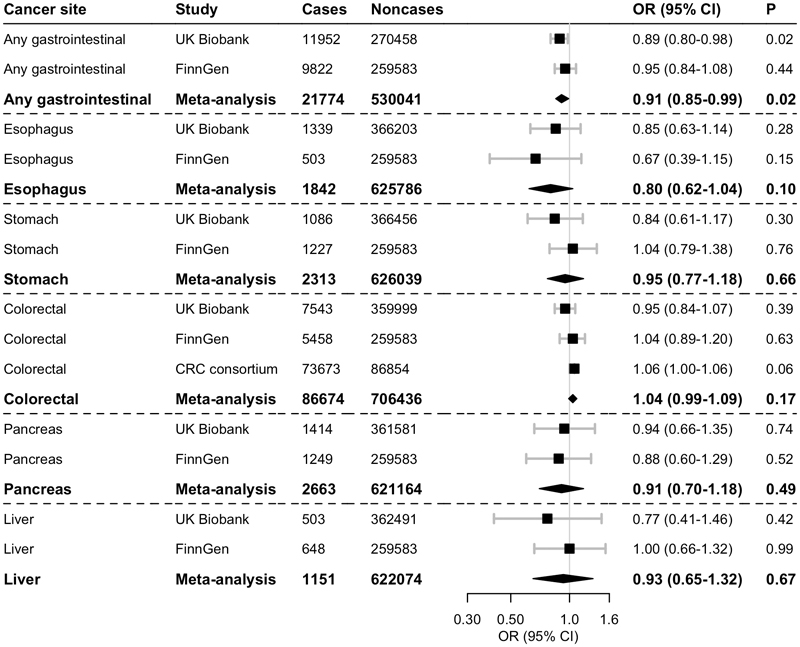
Associations of plasma ghrelin with gastrointestinal cancer risk in two-sample MR analysis using the primary instrument with four genetic variants. CI, confidence interval; CRC, colorectal cancer; OR, odds ratio. ORs are scaled per approximate doubling of genetically predicted plasma ghrelin. There was evidence of modest heterogeneity in the meta-analysis of colorectal cancer (*I*^2^=19%), but no heterogeneity between estimates in meta-analyses of the other cancers (*I*^2^=0%).

Higher plasma ghrelin levels proxied by the four genetic variant instruments were associated with a statistically significant reduction in the risk of gastrointestinal cancer in the UK Biobank and in a meta-analysis of the two studies; the association was inverse but non-significant in FinnGen (Figure 1). The odds ratio per approximate doubling of genetically predicted plasma ghrelin was 0.91 (95% confidence interval, 0.85-0.99; *P*=0.02) in the meta-analysis, without evidence of heterogeneity between studies (*I*^2^=0%). No significant association was observed between the genetically predicted plasma ghrelin levels and any specific gastrointestinal cancer (Figure 1).

The single genetic variant instrument was associated with any gastrointestinal cancer in the UK Biobank but not in FinnGen or in the meta-analysis of both studies (Figure S1). The odds ratio per approximate doubling of genetically predicted plasma ghrelin was 0.89 (95% confidence interval, 0.78-0.98; *P*=0.02) in the UK Biobank and 0.99 (95% confidence interval, 0.93-1.05; *P*=0.62) in the meta-analysis.

### Colocalization analysis

Colocalization analysis provided limited evidence of shared causal variants of plasma ghrelin and gastrointestinal cancer in the UK Biobank at the *GHRL* locus (posterior probability H_4_ = 24.5%) (Table S4 and Figure S2). There was little evidence to suggest the presence of distinct causal variants (posterior probability H_3_ = 2.3%).

## Discussion

This GWAS identified 28 genetic variants that are strongly associated with plasma ghrelin levels. The genetic variants were within ±200 kb of the *GHRL* gene, which encodes preproghrelin, which is post-translationally processed into different peptides, including ghrelin (4,5,32). Our two-sample MR analysis revealed an inverse association between plasma ghrelin proxied by the primary (four SNP) genetic instrument and the risk of gastrointestinal cancer. The association was less robust when a single genetic variant instrument was used, with an inverse association found only in the UK Biobank. There is limited evidence for shared causal variants of plasma ghrelin and gastrointestinal cancer risk at the *GHRL* locus.

Our MR analyses focusing on gastrointestinal cancer were motivated by several previous observational studies that reported inverse associations between the total circulating levels of ghrelin and the risk of gastrointestinal cancers, including esophageal squamous cell carcinoma (9,10), esophageal adenocarcinoma (11), stomach cancer (10,12,13), and colorectal cancer (in the years approaching diagnosis only) (14). Our main MR analysis confirmed a significant inverse association between plasma ghrelin and the composite outcome of gastrointestinal cancer in the UK Biobank study and in a meta-analysis of both studies. The analyses of genetically predicted plasma ghrelin levels in relation to specific gastrointestinal cancers were underpowered, but all associations were in the inverse direction in the UK Biobank and meta-analysis. Colocalization analysis was underpowered, and neither supported nor disproved the existence of shared genetic variants at the *GHRL* locus. The reason for the lack of an association between genetically predicted plasma ghrelin levels and the risk of gastrointestinal cancer in FinnGen is unclear. However, it should be noted that the minor allele frequency (MAF) of the top hit ghrelin-associated SNP (rs34911341) was four times higher in FinnGen (MAF=0.024) than in the Swedish (MAF=0.005) and British (MAF=0.006) populations included in this study as well as in the Icelandic population (MAF=0.008) (33). This difference may explain the discrepancy in the results.

Although most *in vitro* studies have shown that ghrelin promotes tumor development, there are also data showing inhibition of cancer growth and increased apoptosis (3,5–8). Most studies have been conducted on acyl ghrelin, which is a ghrelin isoform that binds to the growth hormone secretagogue receptor and stimulates growth hormone release (1,8). Ghrelin gene can produce bioactive peptides other than ghrelin, primarily des-acyl ghrelin and obestatin, which are generated via alternative splicing or post-translational modifications (32). Although no receptors have been identified for des-acyl ghrelin and obestatin, these peptides have been proven to be active, may either support or antagonize the effect of acyl ghrelin, and may have independent activities (32,34).

The SNP with the strongest association with plasma ghrelin in the present GWAS was also the strongest *cis*-SNP associated with plasma ghrelin (UniProt Q9UBU3) measured with Olink in the UK Biobank (35) and with SomaScan in an Icelandic cohort (33). However, the direction of association of this SNP with plasma ghrelin differed between the methods. The T allele of rs34911341 was positively associated with plasma ghrelin in the UK Biobank (35) (beta coefficients of 1.39 and 1.30 in the discovery and replication samples, respectively; very similar to the estimate in the current GWAS) but negatively associated with plasma ghrelin in the Icelandic study (33). This difference may be related to the fact that the Olink method utilizes two antibodies for the same protein that must simultaneously bind to the protein to provide a signal. In the case of ghrelin, this binding is complicated because the preprotein consists of both ghrelin and obestatin, which act in opposite directions. In general, Olink’s method is considered to have a more reliable protein target specificity and a higher number of phenotypic associations than SomaScan (36).

A strength of this study is the MR design, which diminished the bias due to confounding and reverse causation. Furthermore, the use of a relatively strong primary genetic instrument for exposure and a large number of cases for the composite outcome of gastrointestinal cancer provided a high statistical power in the main MR analysis. Nonetheless, the power was low in the analyses of specific gastrointestinal cancers, except for colorectal cancer, and in the colocalization analysis. Thus, further MR analyses of the association between ghrelin levels and specific gastrointestinal cancers based on large-scale genetic consortia data are warranted. A limitation of this MR study and of previous observational studies on ghrelin and cancer is the inability to separate the effect of acyl and des-acyl ghrelin and obestatin. Another shortcoming is that we were unable to examine the association of plasma ghrelin with the histopathological subtypes of esophageal cancer and molecular subtypes of colorectal cancer. Finally, the study populations comprised individuals of European ancestry, which limits the transferability of our results to non-European populations.

In conclusion, this GWAS identified associations between multiple genetic variations in the *GHRL* gene and plasma ghrelin levels. Our MR analysis provided suggestive evidence in support of a possible causal association between higher plasma ghrelin levels and a reduced risk of gastrointestinal cancer. Further research is warranted to establish the causal role of ghrelin in gastrointestinal cancer prevention.

## Supplementary Material

Supplementary material

## Figures and Tables

**Table 1:** SNPs associated with plasma ghrelin in GWAS analysis of the discovery and replication cohorts and in both cohorts combined

SNP	Chr:position	EA	OA	EAF	Discovery cohort (N=6259)			Replication cohort (N=4483)			Combined cohorts (N=10 742)		
					Beta	SE	*P* value	Beta	SE	*P* value	Beta	SE	*P* value
rs34911341†	3:10331519	T	C	0.005	1.35	0.07	3.07E-76	1.56	0.09	2.83E-68	1.434	0.056	1.03E-142
rs143729751	3:10330266	T	G	0.005	1.24	0.07	9.33E-68	1.47	0.09	2.73E-60	1.328	0.056	5.04E-126
rs150429746	3:10483812	T	C	0.000	1.09	0.08	8.84E-38	NA	NA	NA	1.086	0.085	1.23E-37
rs4684677‡	3:10328453	A	T	0.066	-0.32	0.03	2.02E-23	-0.30	0.04	2.56E-14	-0.316	0.025	6.41E-36
rs56284847‡	3:10512641	T	C	0.051	0.46	0.05	2.60E-17	0.60	0.07	4.69E-18	0.517	0.043	3.93E-33
rs4684676	3:10323108	A	G	0.121	-0.19	0.02	2.53E-16	-0.18	0.03	1.06E-10	-0.186	0.018	2.10E-25
rs2287544	3:10316762	T	C	0.121	-0.19	0.02	3.14E-16	-0.18	0.03	2.32E-10	-0.184	0.018	6.77E-25
rs3774203	3:10317489	A	G	0.121	-0.19	0.02	3.14E-16	-0.18	0.03	2.06E-10	-0.184	0.018	6.77E-25
rs17032621	3:10325638	G	A	0.121	-0.19	0.02	4.12E-16	-0.18	0.03	7.85E-11	-0.186	0.018	2.10E-25
rs715827	3:10311773	G	A	0.121	-0.19	0.02	4.66E-16	-0.18	0.03	2.34E-10	-0.183	0.018	1.21E-24
rs55821288	3:10330822	T	C	0.111	-0.19	0.02	6.10E-16	-0.18	0.03	8.75E-10	-0.185	0.018	7.34E-24
rs34892	3:10525646	A	G	0.939	-0.34	0.04	2.73E-15	-0.35	0.06	3.34E-10	-0.345	0.034	5.97E-24
rs34884	3:10523188	C	T	0.924	-0.33	0.04	2.30E-14	-0.35	0.05	3.72E-11	-0.336	0.033	4.00E-24
rs73026596	3:10271502	C	A	0.116	-0.20	0.03	1.01E-11	-0.18	0.04	1.10E-06	-0.193	0.022	1.40E-18
rs11707451	3:10308772	G	T	0.051	-0.25	0.04	2.70E-10	-0.26	0.05	1.93E-08	-0.257	0.030	2.55E-17
rs173359‡	3:10317277	A	G	0.530	-0.11	0.02	6.53E-10	-0.12	0.02	1.03E-08	-0.112	0.013	3.10E-17
rs4462945	3:10271265	T	C	0.116	-0.20	0.03	1.11E-11	-0.18	0.04	1.12E-06	-0.193	0.023	4.24E-17
rs35682	3:10328782	A	G	0.545	-0.11	0.02	7.98E-10	-0.11	0.02	5.19E-08	-0.109	0.013	5.39E-17
rs168529	3:10314153	G	A	0.530	-0.11	0.02	3.67E-10	-0.12	0.02	4.11E-08	-0.111	0.013	5.88E-17
rs35680	3:10330564	T	C	0.545	-0.11	0.02	1.21E-10	-0.11	0.02	2.28E-07	-0.110	0.013	1.11E-16
rs35683	3:10328250	C	A	0.556	-0.11	0.02	7.64E-10	-0.12	0.02	2.58E-08	-0.110	0.013	1.11E-16
rs1063429	3:10320968	A	T	0.470	0.10	0.02	1.51E-09	0.12	0.02	1.31E-08	0.110	0.013	1.11E-16
rs35681	3:10329377	C	T	0.545	-0.11	0.02	4.61E-10	-0.11	0.02	1.56E-07	-0.108	0.013	3.91E-16
rs171407	3:10326169	A	G	0.571	-0.09	0.02	4.30E-08	-0.12	0.02	3.23E-09	-0.106	0.013	1.34E-15
rs164938	3:10315103	T	G	0.424	-0.10	0.02	1.27E-08	-0.10	0.02	1.54E-06	-0.101	0.014	8.02E-14
rs2241308	3:10295883	A	G	0.222	-0.13	0.02	9.66E-10	-0.11	0.03	2.67E-05	-0.124	0.017	1.78E-13
rs4684040‡	3:10379623	G	A	0.086	0.19	0.04	4.03E-08	0.21	0.04	9.47E-07	0.201	0.028	2.98E-13
rs111796905	3:10210142	A	G	0.126	-0.15	0.03	1.12E-08	-0.11	0.03	1.08E-03	-0.134	0.021	8.88E-11

Abbreviations: Chr, chromosome; EA, effect allele; EAF, effect allele frequency in the discovery cohort; OA, other allele; SE, standard error; SNP, single-nucleotide polymorphism.

* Beta coefficients represent the change in plasma ghrelin levels per additional effect allele. The Olink NPX Manager software was used for data analysis, and a one-unit higher NPX represents an approximate doubling of the measured plasma ghrelin levels.

† The most robust (smallest *P* value) *cis*-SNP was associated with plasma ghrelin and used as a secondary genetic instrument in two-sample MR analyses.

‡ SNPs in low linkage disequilibrium were used as the primary genetic instrument in two-sample MR analyses. These SNPs were selected using clumping in the *TwoSampleMR* package in R with the European population as the reference population.

## Data Availability

Summary statistics for the genetic variants used in this study are shown in Table 1. Data from the UK Biobank is accessible upon application (https://www.ukbiobank.ac.uk/). Data from the FinnGen study is publicly available (https://finngen.gitbook.io/documentation/). Colorectal cancer summary-level data were obtained from a colorectal cancer GWAS consortium (26).

## Abbreviations

GWAS: genome-wide association study
MR: Mendelian randomization
SNP: single-nucleotide polymorphism.

## References

[1] Kojima M, Hosoda H, Date Y, Nakazato M, Matsuo H, Kangawa K (1999). Ghrelin is a growth-hormone-releasing acylated peptide from stomach. Nature.

[2] Zhao CM, Furnes MW, Stenstrom B, Kulseng B, Chen D (2008). Characterization of obestatin- and ghrelin-producing cells in the gastrointestinal tract and pancreas of rats: an immunohistochemical and electron-microscopic study. Cell Tissue Res.

[3] Spiridon IA, Ciobanu DGA, Giusca SE, Caruntu ID (2021). Ghrelin and its role in gastrointestinal tract tumors (Review). Mol Med Rep.

[4] Cui H, Lopez M, Rahmouni K (2017). The cellular and molecular bases of leptin and ghrelin resistance in obesity. Nat Rev Endocrinol.

[5] Seim I, Amorim L, Walpole C, Carter S, Chopin LK, Herington AC (2010). Ghrelin gene-related peptides: multifunctional endocrine / autocrine modulators in health and disease. Clin Exp Pharmacol Physiol.

[6] Chen CY, Asakawa A, Fujimiya M, Lee SD, Inui A (2009). Ghrelin gene products and the regulation of food intake and gut motility. Pharmacol Rev.

[7] Kasprzak A (2022). Role of the Ghrelin System in Colorectal Cancer. Int J Mol Sci.

[8] Kotta AS, Kelling AS, Corleto KA, Sun Y, Giles ED (2022). Ghrelin and Cancer: Examining the Roles of the Ghrelin Axis in Tumor Growth and Progression. Biomolecules.

[9] Murphy G, Kamangar F, Albanes D, Stanczyk FZ, Weinstein SJ, Taylor PR (2012). Serum ghrelin is inversely associated with risk of subsequent oesophageal squamous cell carcinoma. Gut.

[10] Sadjadi A, Yazdanbod A, Lee YY, Boreiri M, Samadi F, Alizadeh BZ (2013). Serum ghrelin; a new surrogate marker of gastric mucosal alterations in upper gastrointestinal carcinogenesis. PLoS One.

[11] de Martel C, Haggerty TD, Corley DA, Vogelman JH, Orentreich N, Parsonnet J (2007). Serum ghrelin levels and risk of subsequent adenocarcinoma of the esophagus. Am J Gastroenterol.

[12] Murphy G, Kamangar F, Dawsey SM, Stanczyk FZ, Weinstein SJ, Taylor PR (2011). The relationship between serum ghrelin and the risk of gastric and esophagogastric junctional adenocarcinomas. J Natl Cancer Inst.

[13] Pritchett NR, Maziarz M, Shu XO, Kamangar F, Dawsey SM, Fan JH (2020). Serum ghrelin and esophageal and gastric cancer in two cohorts in China. Int J Cancer.

[14] Murphy G, Cross AJ, Dawsey SM, Stanczyk FZ, Kamangar F, Weinstein SJ (2018). Serum ghrelin is associated with risk of colorectal adenocarcinomas in the ATBC study. Gut.

[15] Sundkvist A, Myte R, Palmqvist R, Harlid S, Van Guelpen B (2019). Plasma ghrelin is probably not a useful biomarker for risk prediction or early detection of colorectal cancer. Gut.

[16] Boden S, Harbs J, Sundkvist A, Fuchs K, Myte R, Gylling B (2023). Plasma Concentrations of Gut Hormones Acyl Ghrelin and Peptide YY and Subsequent Risk of Colorectal Cancer and Molecular Tumor Subtypes. Cancer Prev Res (Phila).

[17] Assarsson E, Lundberg M, Holmquist G, Bjorkesten J, Thorsen SB, Ekman D (2014). Homogenous 96-plex PEA immunoassay exhibiting high sensitivity, specificity, and excellent scalability. PLoS One.

[18] Warensjo Lemming E, Byberg L, Stattin K, Ahmad S, Lind L, Elmstahl S (2019). Dietary Pattern Specific Protein Biomarkers for Cardiovascular Disease: A Cross-Sectional Study in 2 Independent Cohorts. J Am Heart Assoc.

[19] Larsson SC, Michaelsson K, Mola-Caminal M, Hoijer J, Mantzoros CS (2022). Genome-wide association and Mendelian randomization study of fibroblast growth factor 21 reveals causal associations with hyperlipidemia and possibly NASH. Metabolism.

[20] Marchini J, Howie B (2010). Genotype imputation for genome-wide association studies. Nat Rev Genet.

[21] Hemani G, Zheng J, Elsworth B, Wade KH, Haberland V, Baird D (2018). The MR-Base platform supports systematic causal inference across the human phenome. Elife.

[22] Burgess S, Dudbridge F, Thompson SG (2016). Combining information on multiple instrumental variables in Mendelian randomization: comparison of allele score and summarized data methods. Stat Med.

[23] Larsson SC, Mason AM, Vithayathil M, Carter P, Kar S, Zheng JS (2022). Circulating vitamin C and digestive system cancers: Mendelian randomization study. Clin Nutr.

[24] Grandi NC, Breitling LP, Brenner H Vitamin D and cardiovascular disease: systematic review and meta-analysis of prospective studies. Prev Med.

[25] Kurki MI, Karjalainen J, Palta P, Sipila TP, Kristiansson K, Donner KM (2023). FinnGen provides genetic insights from a well-phenotyped isolated population. Nature.

[26] Fernandez-Rozadilla C, Timofeeva M, Chen Z, Law P, Thomas M, Schmit S (2023). Deciphering colorectal cancer genetics through multi-omic analysis of 100,204 cases and 154,587 controls of European and east Asian ancestries. Nat Genet.

[27] Brion MJ, Shakhbazov K, Visscher PM (2013). Calculating statistical power in Mendelian randomization studies. Int J Epidemiol.

[28] Yavorska OO, Burgess S (2017). MendelianRandomization: an R package for performing Mendelian randomization analyses using summarized data. Int J Epidemiol.

[29] Higgins JP, Thompson SG (2002). Quantifying heterogeneity in a meta-analysis. Stat Med.

[30] Giambartolomei C, Vukcevic D, Schadt EE, Franke L, Hingorani AD, Wallace C (2014). Bayesian test for colocalisation between pairs of genetic association studies using summary statistics. PLoS Genet.

[31] Wallace C (2013). Statistical testing of shared genetic control for potentially related traits. Genet Epidemiol.

[32] Soares JB, Leite-Moreira AF (2008). Ghrelin, des-acyl ghrelin and obestatin: three pieces of the same puzzle. Peptides.

[33] Ferkingstad E, Sulem P, Atlason BA, Sveinbjornsson G, Magnusson MI, Styrmisdottir EL (2021). Large-scale integration of the plasma proteome with genetics and disease. Nat Genet.

[34] Delhanty PJ, Neggers SJ, van der Lely AJ (2012). Mechanisms in endocrinology: Ghrelin: the differences between acyl- and des-acyl ghrelin. Eur J Endocrinol.

[35] Sun BB, Chiou J, Traylor M, Benner C, Hsu Y-H, Richardson TG Genetic regulation of the human plasma proteome in 54,306 UK Biobank participants.

[36] Katz DH, Robbins JM, Deng S, Tahir UA, Bick AG, Pampana A (2022). Proteomic profiling platforms head to head: Leveraging genetics and clinical traits to compare aptamer-and antibody-based methods. Sci Adv.

