# Supplementary material for: Genome-Wide Association and Two-Sample Mendelian Randomization Analyses of Plasma Ghrelin and Gastrointestinal Cancer Risk
Source: Cancer Epidemiol Biomarkers Prev. Author manuscript; Available in PMC 2023 Dec 2. (PMC10690139; doi:10.1158/1055-9965.EPI-23-0757)

**Supplemental Material**

**Genome-Wide Association and Two-Sample Mendelian Randomization Analyses of Plasma Ghrelin and Gastrointestinal Cancer Risk**

| ***Content*** | ***Page*** |
| --- | --- |
| **Table S1.** Classification of each gastrointestinal cancer in UK Biobank and FinnGen | 2 |
| **Table S2.** Power calculations | 3 |
| **Table S3.** Studies included in the colorectal cancer consortium dataset used in the present study | 4 |
| **Table S4.** Colocalization analysis results for plasma ghrelin and gastrointestinal cancer in the UK Biobank at the *GHRL* locus | 8 |
| **Figure S1.** Associations of plasma ghrelin with gastrointestinal cancer risk in two-sample MR analysis using the secondary instrument comprising a single *cis*-SNP | 9 |
|  |  |
| **Figure S2.** Prior and posterior probabilities plots of plasma ghrelin and gastrointestinal cancer in the UK Biobank for genetic variants at the *GHRL* locus | 10 |

**Table S1.** Classification of each gastrointestinal cancer in the UK Biobank and FinnGen

|  | **UK Biobank** | | | |  | **FinnGen** | | |
| --- | --- | --- | --- | --- | --- | --- | --- | --- |
| **Cancer site** | **ICD-9 codes** | **ICD-10 codes** | **Self-report (field 20001)*** | **Cancer histology*** |  | **ICD-9 codes** | **ICD-10 codes** |  |
| Esophagus | 150, V10.03 | C15, Z85.01 | 1017 |  |  | 150 | C15 |  |
| Stomach | 151, V10.04 | C16, Z85.028 | 1018 |  |  | 151 | C16 |  |
| Colorectal | 153, 154.0, 154.1, V10.05, V10.06 | C18, C19, C20, Z85.038, Z85.048 | 1020, 1022, 1023 |  |  | 153, 154 | C18, C19, C20 |  |
| Pancreas | 157 | C25, Z85.07 | 1026 |  |  | 157 | C25 |  |
| Liver | 155.0 | C22.0 | 1024 | 8170, 8171, 8172, 8173, 8174, 8175 |  | 155 | C22 |  |

*The self-report and cancer histology columns provide the internal UK Biobank codes used to define each outcome (available at <https://biobank.ctsu.ox.ac.uk/crystal/coding.cgi?id=3> and https://biobank.ctsu.ox.ac.uk/crystal/coding.cgi?id=38). ICD, international classification of diseases.

**Table S2.** Power calculations

|  |  |  |  |  | **Statistical power at different odds ratios*** | | | | | |
| --- | --- | --- | --- | --- | --- | --- | --- | --- | --- | --- |
| **Cancer site** | **Study** | **Cases** | **Controls** | **Total** | **0.5** | **0.8** | **0.9** | **1.1** | **1.2** | **1.5** |
| Any gastrointestinal | UK Biobank | 11952 | 270458 | 282410 | 100% | 100% | 63% | 63% | 100% | 100% |
|  | FinnGen | 9822 | 259583 | 269405 | 100% | 99% | 55% | 55% | 99% | 100% |
|  |  |  |  |  |  |  |  |  |  |  |
| Esophagus | UK Biobank | 1339 | 366203 | 367542 | 98% | 35% | 12% | 12% | 35% | 97% |
|  | FinnGen | 503 | 259583 | 260086 | 66% | 16% | 8% | 8% | 16% | 66% |
|  |  |  |  |  |  |  |  |  |  |  |
| Stomach | UK Biobank | 1086 | 366456 | 367542 | 94% | 29% | 11% | 11% | 29% | 94% |
|  | FinnGen | 1227 | 259583 | 260810 | 96% | 32% | 12% | 13% | 32% | 96% |
|  |  |  |  |  |  |  |  |  |  |  |
| Colorectum | UK Biobank | 7543 | 359999 | 367542 | 96% | 100% | 45% | 45% | 100% | 96% |
|  | FinnGen | 5458 | 259583 | 265041 | 100% | 88% | 35% | 35% | 88% | 100% |
|  | CRC consortium | 73673 | 86854 | 160527 | 100% | 100% | 99% | 98% | 100% | 100% |
|  |  |  |  |  |  |  |  |  |  |  |
| Pancreas | UK Biobank | 1414 | 361581 | 367542 | 98% | 36% | 13% | 13% | 36% | 98% |
|  | FinnGen | 1249 | 259583 | 260832 | 97% | 33% | 12% | 12% | 33% | 97% |
|  |  |  |  |  |  |  |  |  |  |  |
| Liver | UK Biobank | 503 | 362491 | 367542 | 68% | 16% | 8% | 8% | 16% | 68% |
|  | FinnGen | 648 | 259583 | 260231 | 78% | 19% | 8% | 8% | 19% | 78% |

*Calculations were based on number of cases, total sample size, a phenotypic variance explained by the genetic instrument of 4.6%,

and alpha (type I error rate) of 0.05. Calculations were conducted using an online tool (<https://shiny.cnsgenomics.com/mRnd/>).

**Table S3.** Studies included in the colorectal cancer consortium dataset used in the present study

| **Analytical unit** | **N cases** | **N controls** | **Sub-study** | **Study name** | **Country/Continent** |
| --- | --- | --- | --- | --- | --- |
| **GECCO_OmniExpressExome** | 4 439 | 4 115 |  |  |  |
|  |  |  | COLON | Colorectal Cancer: Longitudinal Observational study on Nutritional and lifestyle factors that influence colorectal tumor recurrence, survival and quality of life | Netherlands |
|  |  |  | DACHS_3 | Darmkrebs: Chancen der Verhütung durch Screening Study | Germany |
|  |  |  | EPIC | European Prospective Investigation into Cancer and Nutrition | Europe |
|  |  |  | HPFS_4 | Health Professionals Follow-Up Study | USA |
|  |  |  | NHS_4 | Nurses’ Health Study | USA |
| **CORECT_EUR_pooled_data_set** | 19 948 | 12 124 |  |  |  |
|  |  |  | ATBC | Alpha-Tocopherol, Beta Carotene Cancer Prevention Study | Finland |
|  |  |  | CCFR_3 | Colon Cancer Family Registry | USA, Canada, Australia |
|  |  |  | CCFR_4 | Colon Cancer Family Registry | USA, Canada, Australia |
|  |  |  | ColoCare_Heidelberg | ColoCare Consortium | Germany |
|  |  |  | ColoCare_Seattle | ColoCare Consortium | USA |
|  |  |  | CPSII_1 | American Cancer Society Cancer Prevention Study II nested case-control study | USA |
|  |  |  | CRCGEN | Colorectal Cancer Genetics & Genomics, Spanish study | Spain |
|  |  |  | ESTHER_VERDI | Epidemiologische Studie zu Chancen der Verhütung, Früherkennung und optimierten Therapie chronischer Erkrankungen in der älteren Bevölkerung; Verlauf der diagnotischen Abklärung bei Krebspatienten | Germany |
|  |  |  | Kentucky | Kentucky Case-Control Study | USA |
|  |  |  | MCCS | Melbourne Collaborative Cohort Study | Australia |
|  |  |  | MEC_2 | Multiethnic Cohort Study | USA |
|  |  |  | MECC_1 | Molecular Epidemiology of Colorectal Cancer Study | Israel |
|  |  |  | MECC_2 | Molecular Epidemiology of Colorectal Cancer Study | Israel |
|  |  |  | MECC_3 | Molecular Epidemiology of Colorectal Cancer Study | Israel |
|  |  |  | MSKCC | Memorial Sloan Kettering Cancer Center Cohort | USA |
|  |  |  | NFCCR | Newfoundland Case-Control Study | Canada |
|  |  |  | NGCCS | PopGen Biobank | Germany |
|  |  |  | NHSII | Nurses’ Health Study | USA |
|  |  |  | SEARCH | Studies of Epidemiology and Risk Factors in Cancer Heredity | UK |
|  |  |  | SLRCCS | Swedish Low-Risk Colorectal Cancer Study | Sweden |
|  |  |  | SMC_COSM | Swedish Mammography Cohort and Cohort of Swedish Men | Sweden |
|  |  |  | USC_HRT_CRC | Los Angeles County Cancer Surveillance Program | USA |
| **CORSA_1** | 1 460 | 774 |  | Colorectal Cancer Study of Austria | Austria |
| **GECCO_Oncoarray_custom_iSelect** | 11 835 | 11 856 |  |  |  |
|  |  |  | CLUEII | Campaign against Cancer and Heart Disease II | USA |
|  |  |  | CORSA_2 | Colorectal Cancer Study of Austria | Austria |
|  |  |  | CPSII_2 | American Cancer Society Cancer Prevention Study II nested case-control study | USA |
|  |  |  | Czech | Czech Republic CCS | Czech Republic |
|  |  |  | EDRN | Early Detection Research Network | USA |
|  |  |  | EPICOLON | EPICOLON | Spain |
|  |  |  | HawaiiCCS_AD | Hawaii Adenoma Study | USA |
|  |  |  | LCCS | Leeds Colorectal Cancer Study | UK |
|  |  |  | NCCCSI | North Carolina Colon Cancer Study, I | USA |
|  |  |  | NCCCSII | North Carolina Colon Cancer Study, II | USA |
|  |  |  | NHS_5_AD | Nurses’ Health Study | USA |
|  |  |  | NSHDS | The Northern Sweden Health and Disease Study | Sweden |
|  |  |  | OSUMC | Columbus-area HNPCC study, Ohio Colorectal Cancer Prevention Initiative, Ohio State University Medical Center | USA |
|  |  |  | PLCO_4_AD | Prostate, Lung, Colorectal, and Ovarian Cancer Screening Trial | USA |
|  |  |  | SELECT | Selenium and Vitamin E Prevention Trial | USA |
|  |  |  | SMS_AD | Screening Markers for Colorectal Cancer Study (advanced adenomas) | USA |
|  |  |  | WHI_3 | Women’s Health Initiative Study | USA |
| **GECCO_stage_1_pooled_data_set** | 11 895 | 14 659 |  |  |  |
|  |  |  | ASTERISK | Association STudy Evaluating RISK for sporadic colorectal cancer | France |
|  |  |  | CCFR_1 | Colon Cancer Family Registry | USA, Canada, Australia |
|  |  |  | CCFR_2 | Colon Cancer Family Registry | USA, Canada, Australia |
|  |  |  | Colo2&3 | Hawai’i Colorectal Cancer Studies 2&3 | USA |
|  |  |  | DACHS_1 | Darmkrebs: Chancen der Verhütung durch Screening | Germany |
|  |  |  | DACHS_2 | Darmkrebs: Chancen der Verhütung durch Screening | Germany |
|  |  |  | DALS_1 | Diet, Activity and Lifestyle Study | USA |
|  |  |  | DALS_2 | Diet, Activity and Lifestyle Study | USA |
|  |  |  | HPFS_1 | Health Professionals Follow-Up Study | USA |
|  |  |  | HPFS_2 | Health Professionals Follow-Up Study | USA |
|  |  |  | HPFS_3_AD | Health Professionals Follow-Up Study | USA |
|  |  |  | MEC_1 | Multiethnic Cohort Study | USA |
|  |  |  | NHS_1 | Nurses’ Health Study | USA |
|  |  |  | NHS_2 | Nurses’ Health Study | USA |
|  |  |  | NHS_3_AD | Nurses’ Health Study | USA |
|  |  |  | OFCCR | Ontario Familial Colorectal Cancer Registry | Canada |
|  |  |  | PHS | Physicians’ Health Study | USA |
|  |  |  | PLCO_1 | Prostate, Lung, Colorectal, and Ovarian Cancer Screening Trial | USA |
|  |  |  | PLCO_2 | Prostate, Lung, Colorectal, and Ovarian Cancer Screening Trial | USA |
|  |  |  | PMH-CCFR | Postmenopausal Hormones Supplementary Study to the Colon Cancer Family Registry | USA |
|  |  |  | VITAL | VITamins And Lifestyle | USA |
|  |  |  | WHI_1 | Women’s Health Initiative Study | USA |
|  |  |  | WHI_2 | Women’s Health Initiative Study | USA |
| **COIN** | 1 950 | 2 162 |  | COIN and COIN-B clinical trial | UK |
| **Croatia** | 689 | 441 |  | Croatia case-control CRC study | Croatia |
| **DACHS 4** | 1 028 | 661 |  | Darmkrebs: Chancen der Verhütung durch Screening | Germany |
| **FIN2** | 1 760 | 14 132 |  | Finnish colorectal cancer collection | Finland |
| **NSCCG** | 6 596 | 7 205 |  | National Study of Colorectal Cancer Genetics | UK |
| **SCOT** | 2 910 | 4 095 |  | Short Course Oncology Treatment (SCOT) trial | UK |
| **ScotlandPhase1** | 932 | 943 |  | Colorectal Cancer Genetic Susceptibility (COGS) study | Scotland |
| **SOCCS_GS** | 4 551 | 8 804 |  | Study Of Colorectal Cancer in Scotland (cases), Generation Scotland (controls) | Scotland |
| **SOCCS_LBC** | 996 | 1 297 |  | Study Of Colorectal Cancer in Scotland (cases), Lothian Birth Cohort (controls) | Scotland |
| **UK1** | 890 | 900 |  | Colorectal Tumour Gene Identification (CoRGI) study | UK |
| **VQ58** | 1 794 | 2 686 |  | VICTOR and QUASAR2 clinical trials (cases), 1958 birth cohort (controls) | UK |

**Table S4.** Colocalization analysis results for plasma ghrelin and gastrointestinal cancer in the UK Biobank at the *GHRL* locus

| **H_0_** | **H_1_** | **H_2_** | **H_3_** | **H_4_** |
| --- | --- | --- | --- | --- |
| 1.26×10^-118^ | 7.32×10^-1^ | 3.99×10^-120^ | 2.30×10^-2^ | 2.45×10^-1^ |

H_0_-H_4_: posterior probabilities of the associations between the two phenotypes examined (i.e., ghrelin and gastrointestinal cancer), evaluating five different conformations:

- H_0_: Neither phenotype has causal genetic variants in the region
- H_1_: The first phenotype (i.e., ghrelin) has a causal genetic variant in the region but the second does not
- H_2_: The second phenotype (i.e., gastrointestinal cancer) has a causal genetic variant in the region but the first does not
- H_3_: Both phenotypes have a causal but distinct genetic variant in the region
- H_4_: The two phenotypes have a shared causal genetic variant in the region

**Figure S1.** Associations of plasma ghrelin with gastrointestinal cancer risk in two-sample MR analysis using the secondary instrument comprising the single *cis*-SNP


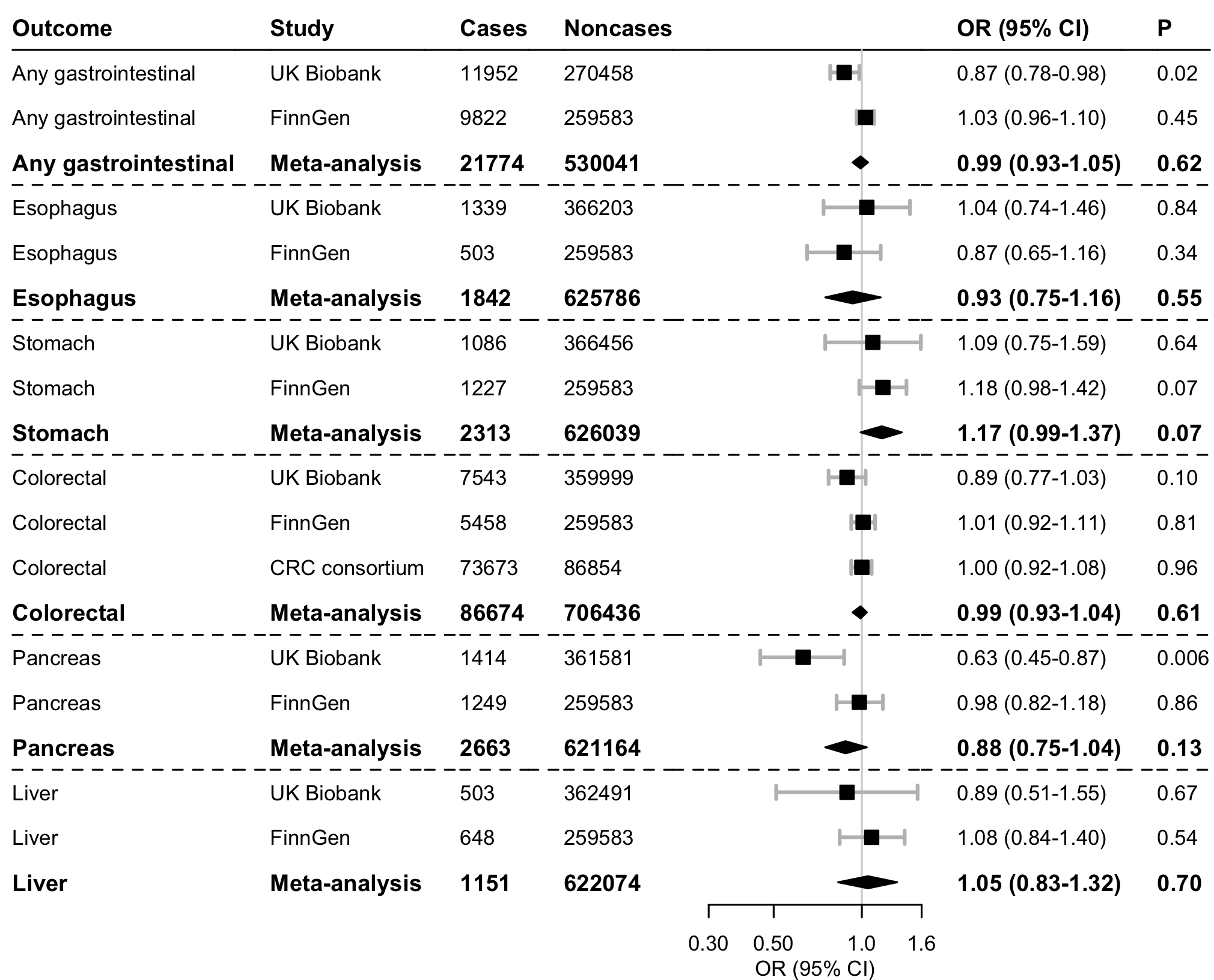


CI, confidence interval; CRC colorectal cancer; OR, odds ratio. ORs are scaled per approximate doubling of genetically predicted plasma ghrelin. Significant heterogeneity between estimates from the two studies was observed in the meta-analyses of any gastrointestinal cancer (*I*^2^=81%), colorectal cancer (*I*^2^=56%), and pancreatic cancer (*I*^2^=82%), but not for esophageal, stomach, and liver cancer (all *I*^2^=0%).

**Figure S2.** Prior and posterior probabilities plots of plasma ghrelin and gastrointestinal cancer in the UK Biobank for genetic variants at the *GHRL* locus


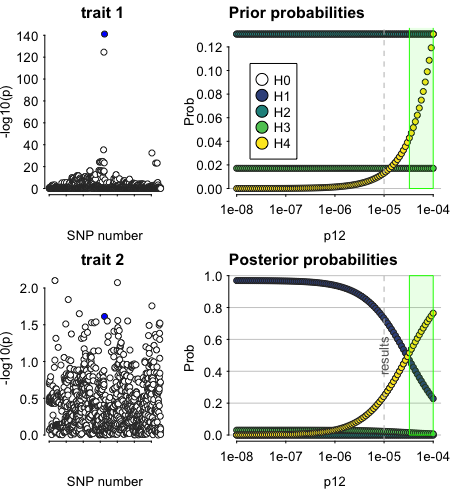

Supplement: Supplementary material [file EMS190944-supplement-Supplementary_material.docx]
