# Supplementary material for: Genome-Wide Association and Two-Sample Mendelian Randomization Analyses of Plasma Ghrelin and Gastrointestinal Cancer Risk
Source: Cancer Epidemiol Biomarkers Prev. 2023 Oct 4;32(12):1771–6. doi: 10.1158/1055-9965.EPI-23-0757 (PMC10690139; doi:10.1158/1055-9965.EPI-23-0757)
Supplement: Supplementary file 1 [file epi-23-0757_figure_s1_suppsf1.docx]

**Figure S1.** Associations of plasma ghrelin with gastrointestinal cancer risk in two-sample MR analysis using the secondary instrument comprising the single *cis*-SNP
