# Supplementary material for: Genome-Wide Association and Two-Sample Mendelian Randomization Analyses of Plasma Ghrelin and Gastrointestinal Cancer Risk
Source: Cancer Epidemiol Biomarkers Prev. 2023 Oct 4;32(12):1771–6. doi: 10.1158/1055-9965.EPI-23-0757 (PMC10690139; doi:10.1158/1055-9965.EPI-23-0757)
Supplement: Supplementary file 3 [file epi-23-0757_table_s1_suppst1.docx]

**Table S1.** Classification of each gastrointestinal cancer in the UK Biobank and FinnGen 

|  | **UK Biobank** |  |  |  | **FinnGen** |  |
| --- | --- | --- | --- | --- | --- | --- |
| **Cancer site** | **ICD-9 codes** | **ICD-10 codes** | **Self-report (field 20001)*** | **Cancer histology*** | **ICD-9 codes** | **ICD-10 codes** |
| Esophagus | 150, V10.03 | C15, Z85.01 | 1017 |  | 150 | C15 |
| Stomach | 151, V10.04 | C16, Z85.028 | 1018 |  | 151 | C16 |
| Colorectal | 153, 154.0, 154.1, V10.05, V10.06 | C18, C19, C20, Z85.038, Z85.048 | 1020, 1022, 1023 |  | 153, 154 | C18, C19, C20 |
| Pancreas | 157 | C25, Z85.07 | 1026 |  | 157 | C25 |
| Liver | 155.0 | C22.0 | 1024 | 8170, 8171, 8172, 8173, 8174, 8175 | 155 | C22 |

*The self-report and cancer histology columns provide the internal UK Biobank codes used to define each outcome (available at [https://biobank.ctsu.ox.ac.uk/crystal/coding.cgi?id=3](https://biobank.ctsu.ox.ac.uk/crystal/coding.cgi?id=3) and https://biobank.ctsu.ox.ac.uk/crystal/coding.cgi?id=38). ICD, international classification of diseases.
