# Supplementary material for: Genome-Wide Association and Two-Sample Mendelian Randomization Analyses of Plasma Ghrelin and Gastrointestinal Cancer Risk
Source: Cancer Epidemiol Biomarkers Prev. 2023 Oct 4;32(12):1771–6. doi: 10.1158/1055-9965.EPI-23-0757 (PMC10690139; doi:10.1158/1055-9965.EPI-23-0757)
Supplement: Supplementary file 4 [file epi-23-0757_table_s2_suppst2.docx]

**Table S2.** Power calculations

|  |  |  |  |  | **Statistical power at different odds ratios*** |  |  |  |  |  |
| --- | --- | --- | --- | --- | --- | --- | --- | --- | --- | --- |
| **Cancer site** | **Study** | **Cases** | **Controls** | **Total** | **0.5** | **0.8** | **0.9** | **1.1** | **1.2** | **1.5** |
| Any gastrointestinal | UK Biobank | 11952 | 270458 | 282410 | 100% | 100% | 63% | 63% | 100% | 100% |
|  | FinnGen | 9822 | 259583 | 269405 | 100% | 99% | 55% | 55% | 99% | 100% |
|  |  |  |  |  |  |  |  |  |  |  |
| Esophagus | UK Biobank | 1339 | 366203 | 367542 | 98% | 35% | 12% | 12% | 35% | 97% |
|  | FinnGen | 503 | 259583 | 260086 | 66% | 16% | 8% | 8% | 16% | 66% |
|  |  |  |  |  |  |  |  |  |  |  |
| Stomach | UK Biobank | 1086 | 366456 | 367542 | 94% | 29% | 11% | 11% | 29% | 94% |
|  | FinnGen | 1227 | 259583 | 260810 | 96% | 32% | 12% | 13% | 32% | 96% |
|  |  |  |  |  |  |  |  |  |  |  |
| Colorectum | UK Biobank | 7543 | 359999 | 367542 | 96% | 100% | 45% | 45% | 100% | 96% |
|  | FinnGen | 5458 | 259583 | 265041 | 100% | 88% | 35% | 35% | 88% | 100% |
|  | CRC consortium | 73673 | 86854 | 160527 | 100% | 100% | 99% | 98% | 100% | 100% |
|  |  |  |  |  |  |  |  |  |  |  |
| Pancreas | UK Biobank | 1414 | 361581 | 367542 | 98% | 36% | 13% | 13% | 36% | 98% |
|  | FinnGen | 1249 | 259583 | 260832 | 97% | 33% | 12% | 12% | 33% | 97% |
|  |  |  |  |  |  |  |  |  |  |  |
| Liver | UK Biobank | 503 | 362491 | 367542 | 68% | 16% | 8% | 8% | 16% | 68% |
|  | FinnGen | 648 | 259583 | 260231 | 78% | 19% | 8% | 8% | 19% | 78% |

*Calculations were based on number of cases, total sample size, a phenotypic variance explained by the genetic instrument of 4.6%, 

and alpha (type I error rate) of 0.05. Calculations were conducted using an online tool ([https://shiny.cnsgenomics.com/mRnd/](https://shiny.cnsgenomics.com/mRnd/)).
