# Supplementary material for: Genome-Wide Association and Two-Sample Mendelian Randomization Analyses of Plasma Ghrelin and Gastrointestinal Cancer Risk
Source: Cancer Epidemiol Biomarkers Prev. 2023 Oct 4;32(12):1771–6. doi: 10.1158/1055-9965.EPI-23-0757 (PMC10690139; doi:10.1158/1055-9965.EPI-23-0757)
Supplement: Supplementary file 5 [file epi-23-0757_table_s3_suppst3.docx]

**Table S3.** Studies included in the colorectal cancer consortium dataset used in the present study

| **Analytical unit** | **N cases** | **N controls** | **Sub-study** | **Study name** | **Country/Continent** |
| --- | --- | --- | --- | --- | --- |
| **GECCO_OmniExpressExome** | 4 439 | 4 115 |  |  |  |
|  |  |  | COLON | Colorectal Cancer: Longitudinal Observational study on Nutritional and lifestyle factors that influence colorectal tumor recurrence, survival and quality of life | Netherlands |
|  |  |  | DACHS_3 | Darmkrebs: Chancen der Verhütung durch Screening Study | Germany |
|  |  |  | EPIC | European Prospective Investigation into Cancer and Nutrition | Europe |
|  |  |  | HPFS_4 | Health Professionals Follow-Up Study | USA |
|  |  |  | NHS_4 | Nurses’ Health Study | USA |
| **CORECT_EUR_pooled_data_set** | 19 948 | 12 124 |  |  |  |
|  |  |  | ATBC | Alpha-Tocopherol, Beta Carotene Cancer Prevention Study | Finland |
|  |  |  | CCFR_3 | Colon Cancer Family Registry | USA, Canada, Australia |
|  |  |  | CCFR_4 | Colon Cancer Family Registry | USA, Canada, Australia |
|  |  |  | ColoCare_Heidelberg | ColoCare Consortium | Germany |
|  |  |  | ColoCare_Seattle | ColoCare Consortium | USA |
|  |  |  | CPSII_1 | American Cancer Society Cancer Prevention Study II nested case-control study | USA |
|  |  |  | CRCGEN | Colorectal Cancer Genetics & Genomics, Spanish study | Spain |
|  |  |  | ESTHER_VERDI | Epidemiologische Studie zu Chancen der Verhütung, Früherkennung und optimierten Therapie chronischer Erkrankungen in der älteren Bevölkerung; Verlauf der diagnotischen Abklärung bei Krebspatienten | Germany |
|  |  |  | Kentucky | Kentucky Case-Control Study | USA |
|  |  |  | MCCS | Melbourne Collaborative Cohort Study | Australia |
|  |  |  | MEC_2 | Multiethnic Cohort Study | USA |
|  |  |  | MECC_1 | Molecular Epidemiology of Colorectal Cancer Study | Israel |
|  |  |  | MECC_2 | Molecular Epidemiology of Colorectal Cancer Study | Israel |
|  |  |  | MECC_3 | Molecular Epidemiology of Colorectal Cancer Study | Israel |
|  |  |  | MSKCC | Memorial Sloan Kettering Cancer Center Cohort | USA |
|  |  |  | NFCCR | Newfoundland Case-Control Study | Canada |
|  |  |  | NGCCS | PopGen Biobank | Germany |
|  |  |  | NHSII | Nurses’ Health Study | USA |
|  |  |  | SEARCH | Studies of Epidemiology and Risk Factors in Cancer Heredity | UK |
|  |  |  | SLRCCS | Swedish Low-Risk Colorectal Cancer Study | Sweden |
|  |  |  | SMC_COSM | Swedish Mammography Cohort and Cohort of Swedish Men | Sweden |
|  |  |  | USC_HRT_CRC | Los Angeles County Cancer Surveillance Program | USA |
| **CORSA_1** | 1 460 | 774 |  | Colorectal Cancer Study of Austria | Austria |
| **GECCO_Oncoarray_custom_iSelect** | 11 835 | 11 856 |  |  |  |
|  |  |  | CLUEII | Campaign against Cancer and Heart Disease II | USA |
|  |  |  | CORSA_2 | Colorectal Cancer Study of Austria | Austria |
|  |  |  | CPSII_2 | American Cancer Society Cancer Prevention Study II nested case-control study | USA |
|  |  |  | Czech | Czech Republic CCS | Czech Republic |
|  |  |  | EDRN | Early Detection Research Network | USA |
|  |  |  | EPICOLON | EPICOLON | Spain |
|  |  |  | HawaiiCCS_AD | Hawaii Adenoma Study | USA |
|  |  |  | LCCS | Leeds Colorectal Cancer Study | UK |
|  |  |  | NCCCSI | North Carolina Colon Cancer Study, I | USA |
|  |  |  | NCCCSII | North Carolina Colon Cancer Study, II | USA |
|  |  |  | NHS_5_AD | Nurses’ Health Study | USA |
|  |  |  | NSHDS | The Northern Sweden Health and Disease Study | Sweden |
|  |  |  | OSUMC | Columbus-area HNPCC study, Ohio Colorectal Cancer Prevention Initiative, Ohio State University Medical Center | USA |
|  |  |  | PLCO_4_AD | Prostate, Lung, Colorectal, and Ovarian Cancer Screening Trial | USA |
|  |  |  | SELECT | Selenium and Vitamin E Prevention Trial | USA |
|  |  |  | SMS_AD | Screening Markers for Colorectal Cancer Study (advanced adenomas) | USA |
|  |  |  | WHI_3 | Women’s Health Initiative Study | USA |
| **GECCO_stage_1_pooled_data_set** | 11 895 | 14 659 |  |  |  |
|  |  |  | ASTERISK | Association STudy Evaluating RISK for sporadic colorectal cancer | France |
|  |  |  | CCFR_1 | Colon Cancer Family Registry | USA, Canada, Australia |
|  |  |  | CCFR_2 | Colon Cancer Family Registry | USA, Canada, Australia |
|  |  |  | Colo2&3 | Hawai’i Colorectal Cancer Studies 2&3 | USA |
|  |  |  | DACHS_1 | Darmkrebs: Chancen der Verhütung durch Screening | Germany |
|  |  |  | DACHS_2 | Darmkrebs: Chancen der Verhütung durch Screening | Germany |
|  |  |  | DALS_1 | Diet, Activity and Lifestyle Study | USA |
|  |  |  | DALS_2 | Diet, Activity and Lifestyle Study | USA |
|  |  |  | HPFS_1 | Health Professionals Follow-Up Study | USA |
|  |  |  | HPFS_2 | Health Professionals Follow-Up Study | USA |
|  |  |  | HPFS_3_AD | Health Professionals Follow-Up Study | USA |
|  |  |  | MEC_1 | Multiethnic Cohort Study | USA |
|  |  |  | NHS_1 | Nurses’ Health Study | USA |
|  |  |  | NHS_2 | Nurses’ Health Study | USA |
|  |  |  | NHS_3_AD | Nurses’ Health Study | USA |
|  |  |  | OFCCR | Ontario Familial Colorectal Cancer Registry | Canada |
|  |  |  | PHS | Physicians’ Health Study | USA |
|  |  |  | PLCO_1 | Prostate, Lung, Colorectal, and Ovarian Cancer Screening Trial | USA |
|  |  |  | PLCO_2 | Prostate, Lung, Colorectal, and Ovarian Cancer Screening Trial | USA |
|  |  |  | PMH-CCFR | Postmenopausal Hormones Supplementary Study to the Colon Cancer Family Registry | USA |
|  |  |  | VITAL | VITamins And Lifestyle | USA |
|  |  |  | WHI_1 | Women’s Health Initiative Study | USA |
|  |  |  | WHI_2 | Women’s Health Initiative Study | USA |
| **COIN** | 1 950 | 2 162 |  | COIN and COIN-B clinical trial | UK |
| **Croatia** | 689 | 441 |  | Croatia case-control CRC study | Croatia |
| **DACHS 4** | 1 028 | 661 |  | Darmkrebs: Chancen der Verhütung durch Screening | Germany |
| **FIN2** | 1 760 | 14 132 |  | Finnish colorectal cancer collection | Finland |
| **NSCCG** | 6 596 | 7 205 |  | National Study of Colorectal Cancer Genetics | UK |
| **SCOT** | 2 910 | 4 095 |  | Short Course Oncology Treatment (SCOT) trial | UK |
| **ScotlandPhase1** | 932 | 943 |  | Colorectal Cancer Genetic Susceptibility (COGS) study | Scotland |
| **SOCCS_GS** | 4 551 | 8 804 |  | Study Of Colorectal Cancer in Scotland (cases), Generation Scotland (controls) | Scotland |
| **SOCCS_LBC** | 996 | 1 297 |  | Study Of Colorectal Cancer in Scotland (cases), Lothian Birth Cohort (controls) | Scotland |
| **UK1** | 890 | 900 |  | Colorectal Tumour Gene Identification (CoRGI) study | UK |
| **VQ58** | 1 794 | 2 686 |  | VICTOR and QUASAR2 clinical trials (cases), 1958 birth cohort (controls) | UK |
