# Supplementary material for: Genome-Wide Association and Two-Sample Mendelian Randomization Analyses of Plasma Ghrelin and Gastrointestinal Cancer Risk
Source: Cancer Epidemiol Biomarkers Prev. 2023 Oct 4;32(12):1771–6. doi: 10.1158/1055-9965.EPI-23-0757 (PMC10690139; doi:10.1158/1055-9965.EPI-23-0757)
Supplement: Supplementary file 6 [file epi-23-0757_table_s4_suppst4.docx]

**Table S4.** Colocalization analysis results for plasma ghrelin and gastrointestinal cancer in the UK Biobank at the *GHRL* locus

| **H**_**0**_ | **H**_**1**_ | **H**_**2**_ | **H**_**3**_ | **H**_**4**_ |
| --- | --- | --- | --- | --- |
| 1.26×10^-118^ | 7.32×10^-1^ | 3.99×10^-120^ | 2.30×10^-2^ | 2.45×10^-1^ |

H_0_-H_4_: posterior probabilities of the associations between the two phenotypes examined (i.e., ghrelin and gastrointestinal cancer), evaluating five different conformations:

- H_0_: Neither phenotype has causal genetic variants in the region

- H_1_: The first phenotype (i.e., ghrelin) has a causal genetic variant in the region but the second does not

- H_2_: The second phenotype (i.e., gastrointestinal cancer) has a causal genetic variant in the region but the first does not

- H_3_: Both phenotypes have a causal but distinct genetic variant in the region

- H_4_: The two phenotypes have a shared causal genetic variant in the region
